# Home Department

**Published:** 1886-10

**Authors:** 


### HOME DEPARTMENT.

**Silver** that is not in frequent use will not tarnish if rubbed in oatmeal.

**Whole cloves** are now used to exterminate the merciless and industrious moth. It is said they are more effectual as a destroying agent than either tobacco, camphor or cedar shavings

**To Keep Insects Out of Bird Cages.**—Tie up a little sulphur in a bag and suspend it in the cage. Red ants, it is said, will never be found in a closet or drawer if a small bag of sulphur be kept in these places.

**To Remove Clinkers.**—To remove clinkers from the stove, sprinkle common table salt on the linings when the stove is cold. Use plenty of it. Build a moderate fire—wood and coal—and in a day or two the clinkers will be gone.

**How to Preserve Eggs.**—To each pailful of water, add two pints of fresh slacked lime and one pint of common salt; mix well. Fill your barrel half full with this fluid, put your eggs down in it any time after June, and they will keep two years if desired.

**A Good Gruel.**—For a sick man's gruel, take our common grits and wash thoroughly. In cooking, use an excess of water, and cook until perfectly soft, pouring off and straining the liquor, which should be salted to the taste. The more water you use, the lighter in body the gruel. If it is desired to make it richer, add about one-half milk; and here you have a splendid gruel for delicate persons and for convalescents. Use the grits from Southern grown corn.

**Sulphur Fumes for Consumption.**—Consumptive patients are advised by a pupil of Liebig, in the *Apotheke Verein*, to live in rooms where one or two drachms of sulphur are melted on a hot stove. The first ten days brings increased cough and irritation, then these cease, and the patient improves rapidly. Persons with catarrh and in early stages of consumption apply to enter chemical factories, where large quantities of sulphur are evaporated daily, and are cured in a few weeks by the inhalations. Cholera and epidemic diseases are never found in such factories.

**The Treatment of Whooping-cough with Illuminating Gas.**—Dr. W. T. Greene (*Med. Press*, April 7, 1886; *Therap. Gaz.*, May, 1886) suggests an easily available improvement on the old plan of sending children on visits to the gas-works. His plan is to attach a piece of rubber tubing to a burner, the tubing being long enough to reach the floor. The gas is turned on just enough to make a perceptible odor, and the child is to inhale it for a few minutes at a time as often as convenient. —*N. Y. Med. Journal.*

**German Sauce.**—One gallon of green tomatoes chopped fine, one quart of celery and one quart of onions, both chopped fine, two gills of white mustard seed, one of ground black pepper, one gill whole allspice, one gill whole cloves, three gills salt, one pound of white sugar, three quarts of good cider vinegar, one gallon chopped cabbage. Put the cabbage and tomatoes under pressure over night, and in the morning add two red peppers chopped fine. Mix all except the spices together and boil until quite tender, stirring often. When done add the spices, stir well and put into your jars.

**To Restore Injured Meat.**—When the brine sours or taints the meat, pour it off, boil it, skim it well, then pour it back again on the meat, boiling hot; this will restore it even when much injured. If tainted meat is immersed in the solution of chloride of lime prescribed for rancid butter, it will restore it. Fresh meat, hams, fish, etc., can be preserved for an indefinite length of time without salt, by a light application of pyroligneous acid applied with a brush; it imparts a fine, smoky flavor to the meat, and is an effectual preservative against its loss.

**Origin of Fruit Canning.**—It is a singular fact that we are indebted to Pompeii for the great industry of canning fruit. Years ago, when the excavations were just beginning, a party of Cincinnatians found in what had been the pantry of a house many jars of preserved figs. One was opened and they were found to be fresh and good. Investigations showed that the figs had been put into jars in a heated state, an aperture left for the steam to escape, and then sealed with wax. The hint was taken, and the next year canning fruit was introduced into the United States, the process being identical with that in vogue in Pompeii twenty centuries ago. The old ladies in America who can tomatoes and peaches do not realize that they are indebted for this art to a people who perished nearly 2,000 years ago.

**How to Keep Cider Sweet.**—Pure sweet cider that is arrested in the process of fermentation before it becomes acetic acid or even alcohol, and with carbonic acid gas worked out, is one of the most delightful beverages. The *Farm, Field and Fireside* recommends the following scientific method of treating cider to preserve its sweetness: When the saccharine matters by fermentation are being converted to alcohol, if a bent tube be inserted air tight into the bung, with the other end into a pail of water, to allow the carbonic acid gas evolved to pass off without admitting any air into the barrel, a beverage will be obtained that is a fit nectar for the gods. A handy way is to fill your cask nearly up to the faucet when the cask is rolled so that the bung is down. Get a common rubber tube and slip it over the end of the plug in the faucet, with the other end in the pail. Then turn the plug so the cider can have communication with the pail. After the water ceases to bubble, bottle or store away.

**Many Practical Purposes for which an Egg Can Be Used.**—For burns and scalds nothing is more soothing than the white of an egg, which may be poured over the wound. It is softer as a varnish than collodion, and, being always at hand, can be applied. It is also more cooling than the sweet oil and cotton which was formerly supposed to be the surest application to allay the smarting pain. It is the contact with the air which gives the extreme discomfort experienced from the ordinary accident of this kind, and anything that excludes the air and prevents inflammation is the thing to be at once applied.

The egg is considered to be one of the best of remedies for dysentery. Beaten up slightly, with or without sugar, and swallowed at a gulp, it tends, by its emollient qualities, to lessen the inflammation of the stomach and intestines, and, by forming a transient coating on these organs, to enable nature to resume her healthful sway over a diseased body. Two, or at most three eggs per day would be all that is required in ordinary cases; and since egg is not merely medicine, but food as well, the lighter the diet otherwise and the quieter the patient is kept, the more certain and rapid is the recovery.

**Brain Work.**—The notion that those who work only with their brain need less food than those who labor with their hands has long been proved to be fallacious. Mental labor causes greater waste of tissue than muscular. According to careful estimates three hours of hard study wear out the body more than a whole day of hard physical exertion. "Without phosphorus, no thought," is a German saying, and the consumption of that essential ingredient of the brain increases in proportion to the amount of labor which this organ is required to perform. The wear and tear of the brain are easily measured by careful examination of the salts in the liquid excretions. The importance of the brain as a working organ is shown by the amount of blood it receives, which is proportionately greater than that of any other part of the body. One-fifth of the blood goes to the brain, though its average weight is only one-fortieth of that of the body. This fact alone would be sufficient to prove that brain workers require more food, and even better food, than mechanics or farm laborers.—*Journal of Chemistry.*

